# Fatal retroperitoneal fungal abscess following surgery for gallbladder cancer: an autopsy case report

**DOI:** 10.1093/jscr/rjag340

**Published:** 2026-05-07

**Authors:** Shuta Tamura, Nanako Hata, Masashi Kimura, Shinnosuke Morikawa, Akira Hida

**Affiliations:** Department of Gastroenterological Surgery, Kochi Health Sciences Center, 2125-1 Ike, Kochi-City, Kochi 781-8555, Japan; Department of Surgery, Matsuyama Shimin Hospital, 2-6-5, Ootemachi, Matsuyama-City, Ehime 790-0067, Japan; Department of Surgery, Matsuyama Shimin Hospital, 2-6-5, Ootemachi, Matsuyama-City, Ehime 790-0067, Japan; Department of Diagnostic Pathology, Matsuyama Shimin Hospital, 2-6-5, Ootemachi, Matsuyama-City, Ehime 790-0067, Japan; Department of Diagnostic Pathology, Matsuyama Shimin Hospital, 2-6-5, Ootemachi, Matsuyama-City, Ehime 790-0067, Japan

**Keywords:** gallbladder cancer, fungal infection, retroperitoneal abscess, *Candida*, postoperative complications, autopsy

## Abstract

Infectious complications after biliary cancer surgery can be severe and fatal. Although fungal infections are uncommon, they have high mortality rates, particularly in older patients, those requiring intensive care, and those receiving prolonged broad-spectrum antibiotics. An 82-year-old man underwent gallbladder bed resection with regional lymphadenectomy for gallbladder cancer. Postoperatively, he experienced massive bleeding requiring repeated transarterial embolization, which caused progressive organ failure. Despite intensive care, including renal replacement therapy and antifungal treatment, the patient developed persistent sepsis and died on postoperative day 29. Drainage fluid cultures revealed *Candida albicans*, and serum β-D-glucan levels were markedly elevated. Autopsy revealed a massive retroperitoneal abscess caused by fungal infection, presumed to be *Candida*, without residual or recurrent malignancy. This case highlights that postoperative fungal infections, although rare, can have catastrophic outcomes following highly invasive biliary surgeries. Early suspicion of fungal infections and prompt intervention are crucial in older patients with multiple risk factors.

## Introduction

Surgical treatment for biliary tract cancer is highly invasive and has a high incidence of postoperative complications, particularly infectious complications. In rare cases, these infections can be severe or fatal. Fungal infections following hepatobiliary surgery have been infrequently reported; however, they are more likely to occur in older patients, those admitted to the intensive care unit (ICU), and those receiving prolonged or multiple courses of broad-spectrum antibiotics [1].

We report a rare and fatal case of a massive retroperitoneal fungal abscess following surgery for gallbladder cancer, in which sepsis due to *Candida* infection was determined to be the cause of death.

## Case report

**Figure 1:**
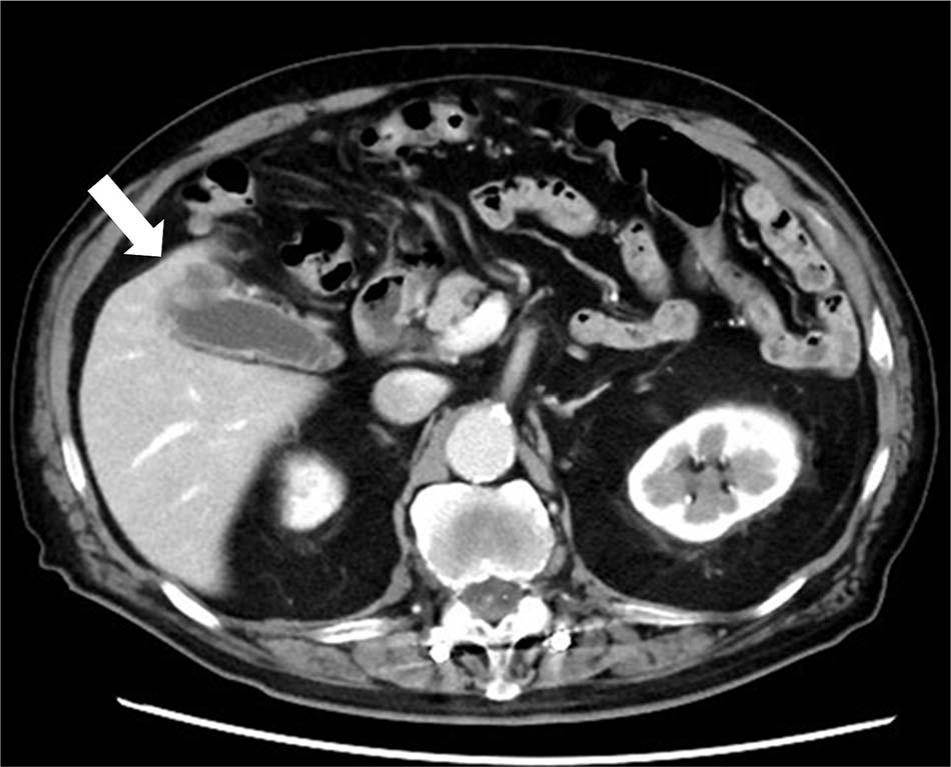
Contrast-enhanced CT showing an irregularly enhanced mass in the gallbladder fundus with suspected partial liver invasion (arrow).

An 82-year-old man was referred after detection of a gallbladder fundus mass on follow-up imaging. His medical history included angina pectoris, transverse colon cancer, femoral fracture, lumbar spinal canal stenosis, and incisional abdominal wall hernia. The case was discussed at a multidisciplinary team meeting including hepatobiliary surgeons, anesthesiologists, radiologists, and intensivists. Dense adhesions were anticipated, given the patient’s surgical history. Laboratory tests were within normal ranges, except for a slightly elevated carcinoembryonic antigen level (5.7 ng/mL). Contrast-enhanced computed tomography (CT) revealed an irregularly enhanced mass in the gallbladder fundus with suspected partial liver invasion (Fig. 1). No distant metastasis was detected. The patient was diagnosed with gallbladder cancer (clinical stage IIIA), and surgical treatment was planned.

## Surgical findings

The operation lasted 7 h 34 min, with blood loss of 3038 ml, and intraoperative transfusion requirement of 8 units of red blood cells and 4 units of fresh frozen plasma. Severe intra-abdominal adhesions were encountered as anticipated, and gallbladder bed resection with regional lymphadenectomy was performed. Final histopathology demonstrated poorly differentiated adenocarcinoma of pT3, pN0, cM0, pStage IIIA, and R0 resection.

## Postoperative course

### PODs 0–2

**Figure 2:**
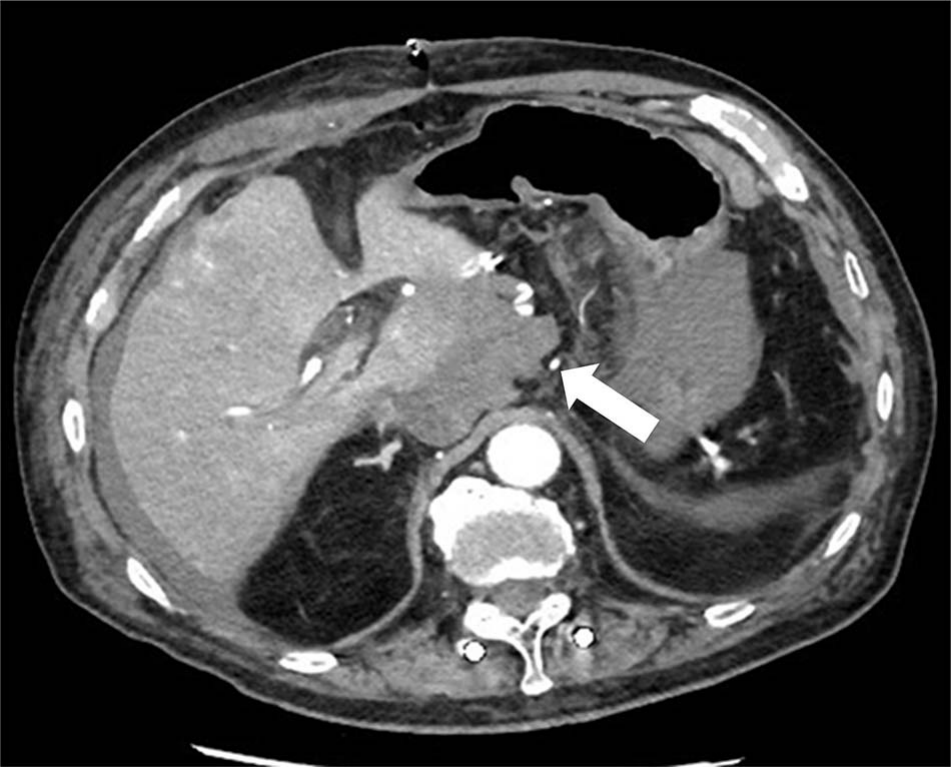
POD 1 contrast-enhanced CT demonstrating a hematoma with active contrast extravasation near the Spiegel lobe (arrow).

The patient was postoperatively admitted to the ICU. Vasopressor support was required for hemodynamic instability. Progressive anemia developed on postoperative day (POD) 1. CT revealed hematoma with contrast extravasation near the Spiegel lobe (Fig. 2), and emergency transarterial embolization (TAE) was performed. Broad-spectrum antibiotics were initiated immediately postoperatively.

### PODs 3–5

**Figure 3:**
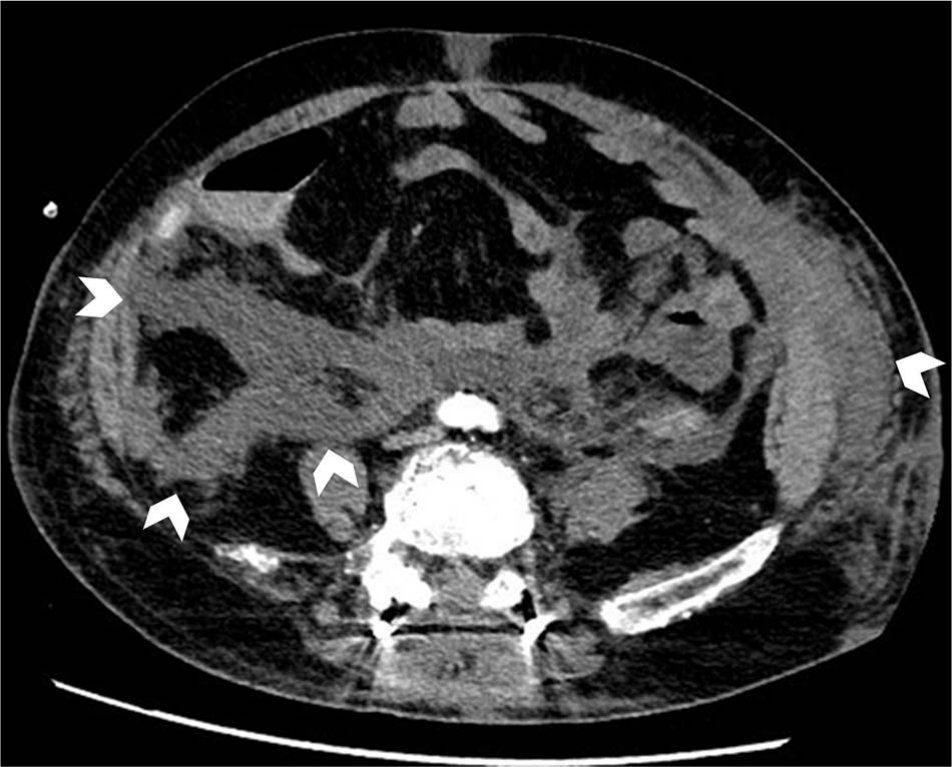
POD 5 CT showing progressive fluid collection in the retroperitoneal space (arrowheads).

On POD 3, oliguria developed with worsening liver and renal function. Continuous hemodiafiltration was initiated. On POD 5, persistent bloody drainage prompted repeat CT, which demonstrated a pseudoaneurysm of the posteroinferior pancreaticoduodenal artery requiring repeat TAE. CT also demonstrated increasing retroperitoneal fluid collection (Fig. 3). At that time, the collection was interpreted as postoperative hematoma rather than abscess, as no rim enhancement or gas formation was evident.

### PODs 6–10

On POD 7, fever and elevated inflammatory markers persisted, β-D-glucan was measured due to suspected fungal infection. The serum β-D-glucan levels were markedly elevated (314.5 pg/mL). Blood cultures subsequently grew *Candida albicans*. Antifungal therapy with micafungin (100 mg/day) was initiated. Transthoracic echocardiography showed no evidence of infective endocarditis. The drainage fluid culture on POD 8 yielded *C. albicans*.

### PODs 11–22

On POD 12, serial CT demonstrated persistence of retroperitoneal fluid without clear encapsulation amenable to safe drainage. Hemodynamic instability and progressive coagulopathy due to liver failure limited invasive source control. The serum PT level was markedly low at 29%. Despite antifungal drug therapy, inflammatory markers remained elevated. Persistent fever prompted escalation to liposomal amphotericin B (300 mg/day) on POD 15.

### PODs 23–29

On POD 23, thrombocytopenia and laboratory findings were consistent with disseminated intravascular coagulation. The serum β-D-glucan levels exceeded 600 pg/mL. Respiratory failure with acute respiratory distress syndrome developed, requiring noninvasive ventilation. Despite maximal supportive care, including with vasopressors and renal replacement therapy, the patient died on POD 29.

## Autopsy findings

**Figure 4:**
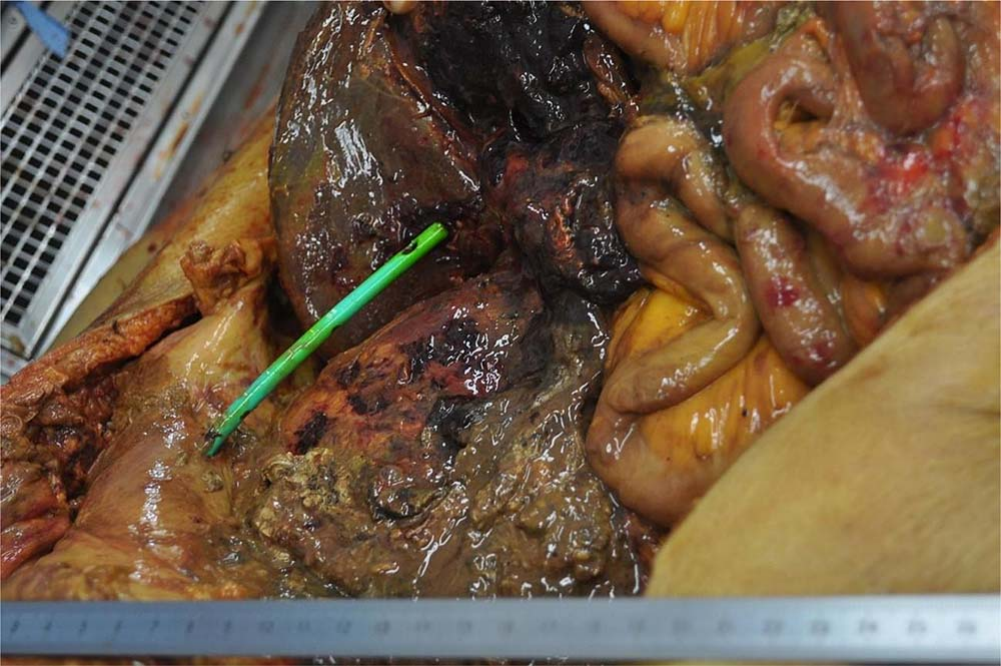
Gross autopsy findings showing a massive abscess in the lower retroperitoneal space.

**Figure 5:**
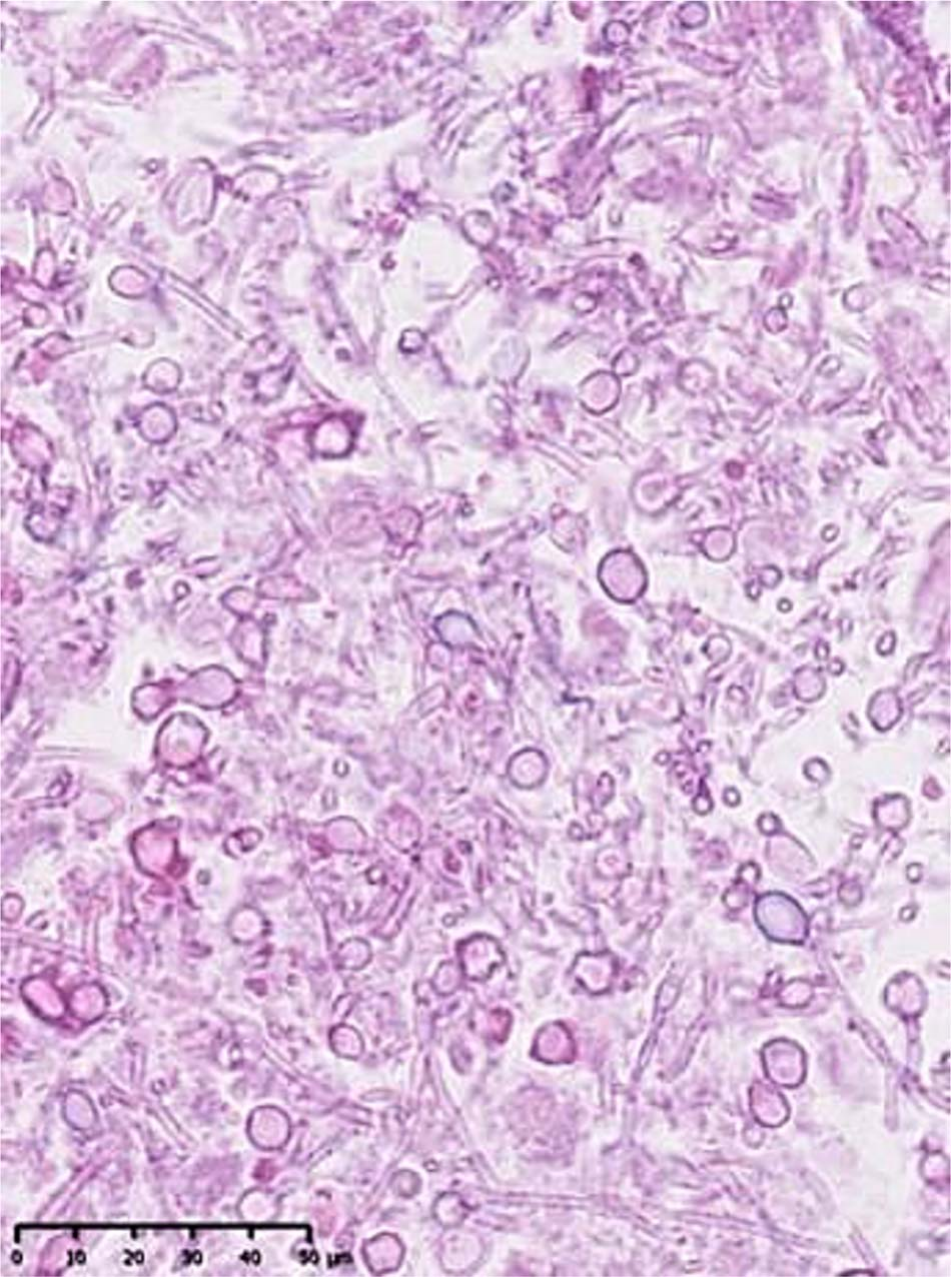
Histopathological examination of the abscess demonstrating filamentous fungal organisms, consistent with *Candida* species (hematoxylin and eosin stain).

Autopsy revealed a massive abscess occupying the lower retroperitoneal space (Fig. 4). Histological examination demonstrated fungal organisms consistent with *Candida* species (Fig. 5). The liver exhibited severe ischemic injury. No residual or recurrent gallbladder cancers were identified. Retrospective review of imaging suggested progressive organization of hematoma that likely evolved into fungal abscess.

## Discussion

Surgery for biliary tract cancer is associated with high morbidity, and infectious complications require particular attention. Although postoperative fungal infections are rare, several reports have documented their occurrence, especially in patients with biliary obstruction or severe inflammation. Postoperative fungal infections remain an important but underrecognized complication of abdominal and hepatobiliary surgery. Previous studies and clinical guidelines have identified advanced age, prolonged ICU stay, extensive exposure to broad-spectrum antibiotics, invasive devices, and postoperative intra-abdominal complications as major risk factors for invasive candidiasis and intra-abdominal fungal infections in surgical patients [1–3].

Lenz *et al.* reported that fungal infections are more likely to occur in the presence of biliary obstruction and are unrelated to oral or fecal fungal colonization or previous endoscopic procedures [4]. Hartmann *et al.* emphasized that fungi are not part of the normal biliary microbiota and that the detection of *Candida* in bile or biliary tissue should be regarded as true infection rather than colonization, particularly in malignant biliary obstruction, which is associated with poor prognosis [5].

Risk factors for postoperative fungal infections include advanced age, prolonged ICU stays, multiple antibiotic use, and central venous catheter placement [1–3], all of which were present in this patient. Additionally, postoperative hemorrhage likely resulted in hematoma formation, which may have served as a nidus for fungal proliferation and subsequent retroperitoneal abscess formation.

Retroperitoneal abscesses often present with nonspecific symptoms and are frequently diagnosed late. Although most cases originate from renal or gastrointestinal sources and involve polymicrobial infections, fungal retroperitoneal abscesses are extremely rare [6]. This case illustrates the complex interplay between hemorrhagic complications and invasive fungal infection following highly invasive hepatobiliary surgery. Repeated postoperative bleeding was managed with transarterial embolization rather than surgical re-exploration owing to severe adhesions, hemodynamic instability, and evolving coagulopathy. Endovascular management was considered the safest option at the time.

Postoperative hematoma likely served as a nidus for fungal proliferation. Although retroperitoneal fluid collection was identified on POD 5, radiologic features were initially consistent with those of hematoma. Early differentiation between hematoma and infected collection remains challenging in the immediate postoperative setting.

Although antifungal therapy was promptly initiated upon detection of candidemia and elevated β-D-glucan, definitive source control was not feasible owing to hemodynamic instability and disseminated intravascular coagulation. This case underscores the critical importance of early recognition and aggressive evaluation of postoperative collections in high-risk patients.

This case raises concerns regarding the surgical indications for older patients with sarcopenia. Although the patient’s preoperative organ function and nutritional status were preserved, the patient had severe sarcopenia, which is known to be associated with higher postoperative morbidity [7]. However, determining surgical ineligibility based solely on these factors remains challenging.

We report a rare and fatal case of a massive retroperitoneal abscess caused by a fungal infection following surgery for gallbladder cancer. Clinicians should maintain a high index of suspicion for postoperative fungal infections in older patients undergoing highly invasive biliary surgery. Early diagnosis and prompt antifungal treatment are critical for improving patient outcomes.
